# *Helicobacter pylori*-Induced Heparanase Promotes *H. pylori* Colonization and Gastritis

**DOI:** 10.3389/fimmu.2021.675747

**Published:** 2021-06-17

**Authors:** Li Tang, Bo Tang, Yuanyuan Lei, Min Yang, Sumin Wang, Shiping Hu, Zhuo Xie, Yaojiang Liu, Israel Vlodavsky, Shiming Yang

**Affiliations:** ^1^ Department of Gastroenterology, Xinqiao Hospital, The Army Medical University, Chongqing, China; ^2^ Technion Integrated Cancer Center (TICC), Rappaport Faculty of Medicine, Technion-Israel Institute of Technology, Haifa, Israel

**Keywords:** *Helicobacter pylori*, heparanase, macrophage, polarization, MAPK

## Abstract

Chronic gastritis caused by *Helicobacter pylori* (*H. pylori*) infection has been widely recognized as the most important risk factor for gastric cancer. Analysis of the interaction between the key participants in gastric mucosal immunity and *H. pylori* infection is expected to provide important insights for the treatment of chronic gastritis and the prevention of gastric cancer. Heparanase is an endoglycosidase that degrades heparan sulfate, resulting in remodeling of the extracellular matrix thereby facilitating the extravasation and migration of immune cells towards sites of inflammation. Heparanase also releases heparan sulfate-bound cytokines and chemokines that further promote directed motility and recruitment of immune cells. Heparanase is highly expressed in a variety of inflammatory conditions and diseases, but its role in chronic gastritis has not been sufficiently explored. In this study, we report that *H. pylori* infection promotes up-regulation of heparanase in gastritis, which in turn facilitates the colonization of *H. pylori* in the gastric mucosa, thereby aggravating gastritis. By sustaining continuous activation, polarization and recruitment of macrophages that supply pro-inflammatory and pro-tumorigenic cytokines (i.e., IL-1, IL-6, IL-1β, TNF-α, MIP-2, iNOS), heparanase participates in the generation of a vicious circle, driven by enhanced NFκB and p38-MAPK signaling, that supports the development and progression of gastric cancer. These results suggest that inhibition of heparanase may block this self-sustaining cycle, and thereby reduce the risk of gastritis and gastric cancer.

## Introduction

*Helicobacter pylori* (*H. pylori*) is a spiral, microaerobic gram-negative bacterium (1). Epidemiologic studies reveal that about 50% of people worldwide are infected with *H. pylori* (2). *H. pylori* infection induces a significant inflammatory response in the gastric mucosa, accompanied by infiltration of immune cells, resulting in chronic infection and sustained damage to gastric mucosal tissues (3, 4). Hence, chronic gastritis caused by *H. pylori* has been widely recognized as the most important risk factor for gastric cancer (5). *H. pylori* infection usually requires antibiotic therapy, but it is increasingly difficult to achieve eradication in some patients, where antibiotics by itself are not sufficient to cure the disease (6). Although the mechanisms of sustained colonization and chronic infection by *H. pylori* in the gastric mucosa are not clear, existing studies suggest that the interaction between gastric epithelial cells and gastric mucosal immune cells is a key feature in the pathogenesis of *H. pylori* infection (7). Therefore, identifying key participants in gastric mucosal immunity and *H. pylori* infection is likely to provide insights that will lead to new treatment modalities of chronic gastritis and prevention of gastric cancer.

By sequestering cytokines and chemokines, mediating the interaction between leukocytes and endothelium in the extracellular matrix (ECM), and facilitating receptor–ligand binding on the surface of immunocytes, heparan sulfate (HS) plays critical roles in multiple inflammatory processes (8). Through degradation and remodeling of the HS polysaccharide chains in the ECM and cell surfaces, heparanase facilitates the extravasation and migration of immune cells towards sites of inflammation (9). Also, heparanase releases HS-bound cytokines and chemokines which further establish concentration gradients that facilitate the bioavailability, activation and directed motility of immune cells (9–11).

It has been previously reported that heparanase is highly expressed in a variety of inflammatory diseases (12, 13), including ulcerative colitis, acute pancreatitis (14, 15), acute vasculitis (16), acute glomerulonephritis (17), hypersensitivity pneumonia (18) and sepsis (19). The role of heparanase in chronic gastritis caused by *H. pylori* has not yet been explored. In this study, we report, for the first time, that *H. pylori* infection promotes the expression of heparanase in gastritis. Heparanase, in turn, further promotes the colonization of *H. pylori* and aggravates gastritis, forming a vicious circle driven by enhanced NFκB and p38-MAPK signaling and the generation of pro-inflammatory and pro-tumorigenic cytokines. These results suggest that inhibition of heparanase will block this self-sustaining cycle, and thereby facilitate the eradication of *H. pylori* and reduce the risk of gastritis and gastric cancer.

## Materials and Methods

### Patients and Specimens

Gastric biopsy specimens were collected from patients who underwent electronic gastroscope for gastritis-related symptoms (i.e., ventosity, rhythmic epigastric pain, dyspepsia) at the endoscopy center of the department of gastroenterology, Xinqiao Hospital, The Army Medical University. Gastritis mucosa tissues (two to four nearby spots) were collected by endoscopic biopsy, and urease tests were performed immediately by using urease test paper (Kedi Tech, Zhuhai, China). The tissue samples were then transferred to labeled frozen pipes and stored in a liquid nitrogen. These samples were used for DNA and RNA extraction, H&E staining, *H. pylori* testing and immunofluorescence. Individuals with atrophic gastritis, hypochlorhydria, antibiotic treatment, autoimmune disease, infectious diseases, and cancer, were excluded. The study was approved by the Ethics Committee of Xinqiao Hospital, The Army Medical University. Written informed consent was obtained from each individual. Demographic characteristics of the patients are presented in **Table 1**.

**Table 1:** Demographic characteristics of enrolled patients.

Characteristics	*H. pylori* negative	*H. pylori* positive	
	Normal tissue	Chronic gastritis	Intestinal metaplasia
Gender			
Male	10 (23.8%)	5 (11.9%)	5 (11.9%)
Female	14 (33.3%)	6 (14.3%)	2 (4.8%)
Age (years old)	29.96 ± 6.36	33.00 ± 6.45	46.57 ± 12.83
Weight (kg)	62.88 ± 5.89	65.00 ± 11.56	66.57 ± 7.55
Height (cm)	163.79 ± 7.80	161.54 ± 11.59	167.00 ± 7.57
Marital status			
Unmarried	8 (19.0%)	3 (7.1%)	2 (4.8%)
Married	16 (38.1%)	8 (19.0%)	5 (11.9%)

### Mice

The heparanase knockout (Hpa-KO) mice have been described previously (20). C57BL/6 mice were purchased from TengXin Bio, Chongqing. All mice used in the experiments were free of pathogenic murine viruses, bacteria and parasites and were fed with sterile food and water in a specific pathogen-free environment. Breeding and animal experiments were performed at the experimental animal center of Xinqiao hospital and were approved by the Ethics Committee of Xinqiao Hospital, The Army Medical University. Mice were euthanized at the 8th week after *H. pylori* infection. The stomach was isolated and the forestomach removed. Stomach contents were washed and cleaned by sterile phosphate buffer saline (PBS). After flattening on a sterile foam plastic board, half of the tissue was cut into four parts for RNA isolation, DNA isolation, protein extraction and tissue fixation for further immunohistochemistry and immunofluorescence analyses.

### *H. pylori* Infection Test

*H. pylori* infection was determined by rapid urease test using biopsy specimens taken from the antrum. Infection was subsequently confirmed by RT-PCR and agarose gel electrophoresis using *H. pylori* specific 16S ribosomal DNA (rDNA) primers. The ‘gold standard’ culture test was applied in the case of two patients, as described in **Supplementary Figure 3**.

### Cells

GES-1 normal human gastric mucosa epithelial cells were purchased from the National Biomedical Laboratory Cell Resource Bank, Beijing, China. Cells were cultured in RPMI-1640 (GE Healthcare Life Sciences, UT, USA) medium supplemented with 10% fetal bovine serum (Gibco, Thermo Fisher Scientific, MA, USA), and penicillin–streptomycin solution (Beyotime Biotech, Beijing) in humidified air containing 5% CO_2_ at 37°C.

### *H. pylori* Strain and Infection of Mice

*H. pylori* PMSS1 [cagA^+^ wild type *H. pylori* prone to colonization in mouse (21)] was kindly provided by Dr. Peng Xie, Department of Gastroenterology, the First Affiliated Hospital of Nanchang University. *H. pylori* strains were grown in agar plate contained campylobacter agar base (Oxiod, #CM0689, Thermo Fisher Scientific, MA, USA) supplemented with 10% sterile defibrinated sheep blood at 37°C under microaerophilic conditions in 5% oxygen, 10% CO_2_ and 85% nitrogen. For infection of mice, *H. pylori* colony on the agar plate were scraped and transfered to culture in Brucella broth containing 5% fetal bovine serum (FBS) and maintained at 37°C under microaerobic conditions with continuous gentle shaking. After culturing for 1–2 days, live bacteria were centrifuged and adjusted to 5 × 10^9^ colony forming units (CFU)/ml. *H. pylori* was administrated to mice by gavage (100 ul of 5 × 10^9^ CFU/mouse), three times at 2-day intervals. Mice were fasted 6 h before each gavage. *H. pylori* infection and *H. pylori*-induced gastritis were evaluated by TaqMan real-time PCR of *H. pylori* 16S rDNA, urease biopsy assay, Warthin–Starry, H&E, and immunohistochemical stainings (22).

### Evaluation of Bacteria Colonization

DNA of the biopsy specimens was extracted with Blood/Cell/Tissue Genomic DNA Extraction Kit (Tiangen Biotech, Beijing). *H. pylori* colonization was quantified by measuring *H. pylori*-specific 16S rDNA using specific primers and the TaqMan method (22). GAPDH (for mouse samples) and human-β-globin (for human samples) were used as an internal control to normalize the PCR reaction. The TaqMan real-time PCR was performed on a StepOne RT-PCR system (Thermo Fisher Scientific, MA, USA) with Premix Ex Taq™ (Probe qPCR) (RR390a, Takara Bio, Dalian) according to the manufacturer’s instructions. Primers for detecting *H. pylori* colonization by using cDNA templates are listed in **Supplementary Table 1**.

### Real-Time PCR

RNA of samples and cultured cells was extracted with RNAiso Plus (9109, Takara Bio, Dalian). RNA was then reversely transcribed into complementary DNA with PrimeScript RT Reagent Kit with gDNA Eraser (RR047a, Takara Bio, Dalian). Real-time PCR was performed on a StepOne RT-PCR system (Thermo Fisher Scientific, MA, USA) with TB Green^®^ Premix Ex Taq™ II (RR820a, Takara Bio, Dalian) according to the manufacturer’s instructions. mRNA was measured using RT-qPCR with the relevant primers. Relative mRNA levels were calculated as relative expression by using 2^−ΔΔCT^ algorithm. Primers for detecting mRNA by using cDNA templates are listed in **Supplementary Table 2**.

### Flow Cytometry

Half of the mouse gastric tissue was used for isolation of single cells, as described (22). Fresh tissues were washed twice with Hanks’ solution containing 1% FBS, cut into small pieces (1 mm^3^), transferred to RPMI 1640 medium (GE Healthcare Life Sciences, UT, USA) containing 1 mg/ml collagenase IV (Thermo Fisher Scientific, MA, USA) and 10 mg/ml DNase I (Sigma-Aldrich, MO, USA), and dissociated mechanically in gentle MACS™ C Tubes (Miltenyi Biotec, CA, USA) using “M intestine 001” program in the gentle MACS™ Dissociator (Miltenyi Biotec, CA, USA). The C tubes were then transferred to a 37°C incubator, incubated for 1 h under continuous rotation and the resulting cell suspensions filtered using a 70-μm cell strainer (BD Labware, NY, USA). The isolated single cells were collected, stained with antibodies and analyzed by flow cytometry. Data were analyzed using flow Jo software (BD, NY, USA).

### Immunohistochemistry

Formaldehyde fixed and paraffin-embedded sections of gastritis tissue samples were heated in a 60°C oven for 2 h, immersed in xylene for 30 min for dewaxing and immersed in increasing concentrations of alcohol for rehydration. Tissue sections were then subjected to antigen retrieval with citrate under medium heat in a microwave oven for 10 min, cooled for 2 min and incubated with 0.5% Triton X-100 for permeabilization. Hydrogen peroxide and 1% BSA were added in sequence for catalase and antigen blocking, respectively. Tissue sections were then incubated (1 h, 37°C) with primary antibodies (i.e., anti-heparanase polyclonal antibody #733, diluted 1:200) (19, 23), washed three times and incubated (30 min) with anti-mouse/rabbit secondary antibodies. Next, DAB chromogenic reagent was added for color development, and hematoxylin was used for counterstaining.

### Immunofluorescence

Fresh frozen sections of gastritis tissue samples were incubated in 100% methanol (chilled at −20°C) at room temperature for 5 min, and then in 4% paraformaldehyde in PBS (pH 7.4) for 10 min at room temperature. Tissue sections were blocked with 1% BSA and glycine in PBST (PBS+ 0.1% Tween 20) for 30 min followed by incubation with the diluted primary antibodies overnight at 4°C. Sections were then incubated with secondary antibodies for 1 h at room temperature, mounted and sealed with coverslips. Photographs were taken with a laser scanning confocal microscope.

### Western Blot Analysis

Western blotting was performed as previously described (23, 24). Briefly, proteins were extracted using RIPA lysis buffer (Beyotime Biotechnology, Beijing, China) containing phenylmethanesulfonyl fluoride (PMSF) (Roche, Basel, Switzerland), and a protein phosphatase inhibitor (Roche, Basel, Switzerland). Thirty micrograms of proteins were subjected to 10% SDS-PAGE and then transferred to a polyvinylidene difluoride (PVDF) membrane (Roche, Rotkreuz, Switzerland). The membrane was blocked with 5% (w/v) skim milk in TBST (20 mM Tris–HCl [pH 8.0], 150 mM NaCl, and 0.1% [v/v] Tween-20) for 2 h and incubated with anti-heparanase (polyclonal antibody #733), anti-iNOS (Santa Cruz Biotechnology, TX, USA), anti-p/NF-κB (Santa Cruz Biotechnology, TX, USA), anti-p/ERK1/2 (Santa Cruz Biotechnology, TX, USA), anti-phospho-p38 (Santa Cruz Biotechnology, TX, USA), anti-GAPHD (Zhongshan Golden Bridge Biotech, Beijing, China) or anti-β-actin (Zhongshan Golden Bridge Biotech, Beijing, China) as primary antibodies. The membrane was washed with TBST, incubated with horseradish peroxidase-conjugated antibody against mouse IgG (1 h at room temperature), and rinsed with TBST. Proteins were visualized with ECL Western Blotting Detection Reagents (GE Healthcare, NJ, USA) and images were analyzed with Quantity One 4.1 software (Bio-Rad). The experiments were repeated at least three times.

### Statistical Analysis

Data were analyzed using R (R Core Team, Vienna, Austria). Mann–Whitney U-test was generally used to analyze the differences between two groups of continuous value. R build-in function wilcox.test() was used to perform Mann–Whitney U-test. All data were analyzed using two-tailed tests. The Chi-square test was used to compare classified disordered data groups. Association between *H. pylori* infection and the expression of heparanase was analyzed by Spearman correlation coefficient. p <0.05 was considered statistically significant.

## Results

### Heparanase Expression Is Increased in *H. pylori*-Induced Chronic Gastritis

**Figure 1:**
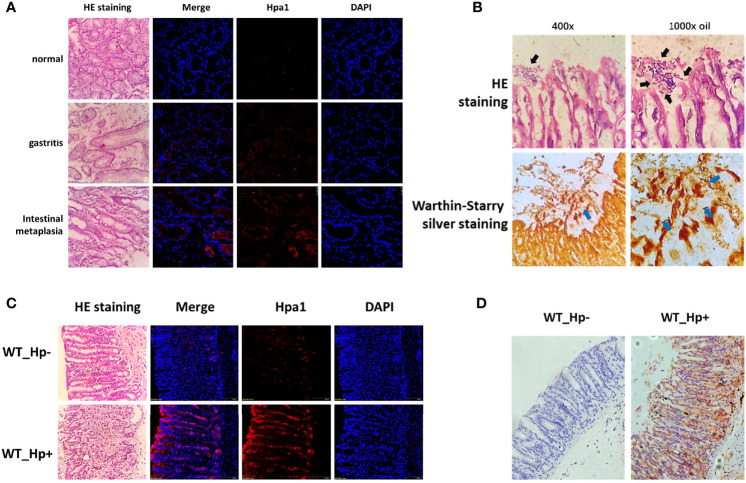
High expression of heparanase in gastric tissue. **(A)** Human gastric tissue biopsies were collected during gastroscopy examination. Normal gastric tissue (gastric antrum), *H. pylori*-infected chronic gastritis tissue, and intestinal metaplasia tissue were included. Left panels: Hematoxylin and eosin (H&E) staining. Right panels: Immunofluorescent staining. Shown are representative images of immunofluorescent staining applying anti-heparanase antibody (red), and nuclear counterstaining (DAPI, blue). **(B)** *H. pylori* strain PMSS1 was used to infect wild type C57BL/6 mice *via* oral gavage, once every 2 days, 5 × 10^9^ CFU each time, for three consecutive times. Eight weeks later, antral gastric mucosa was collected and 5-micron sections were subjected to H&E (upper panels) and Warthin–Starry silver (lower panels) staining, which indicates successful infection of *H. pylori*. Black and blue arrow indicate the colonized helicobacter pylori. **(C)** Immunofluorescent staining of normal mouse gastric tissue and mouse *H. pylori*-infected chronic gastritis tissue. Left panels: Hematoxylin and eosin (H&E) staining. Right panels: immunofluorescent staining. Shown are representative images of staining for heparanase (red), and nuclear counterstaining (DAPI, blue). **(D)** Immunostaining of WT (un-infected) mouse gastric tissue (left panel) and *H. pylori*-infected chronic gastritis tissue (right panel).

To verify the expression of heparanase in chronic gastritis, we obtained gastroscopy biopsy specimens of fresh gastric mucosal tissues from patients who underwent electronic gastroscope for gastritis-related symptoms, including *H. pylori-*positive chronic gastritis mucosal tissues, *H. pylori*-positive chronic atrophic gastritis mucosal tissues, and normal mucosal tissues. Fresh frozen sections were prepared, and the expression of heparanase was examined by immunofluorescent staining using anti-heparanase antibodies. Compared to normal gastric tissue, heparanase staining was readily detected in chronic gastritis, gastric mucosa and chronic atrophic gastric mucosa (**Figure 1A** and **Supplementary Figure 1A**). To study the role of heparanase in gastritis, *H. pylori* strain PMSS1 was used to infect C57BL/6 mice *via* oral gavage, once every 2 days, 5 × 10^9^ CFU each time, for three consecutive times. After 8 weeks, mice were sacrificed, the gastric tissues were collected, and colonization of *H. pylori* in the gastric mucosa was confirmed by H&E and Warthin–Starry silver staining (24) (**Figure 1B**). Notably, heparanase staining intensity was increased prominently in *H. pylori*-infected gastric mucosa *vs.* control, un-infected gastric tissue. This was evident by immunofluorescent staining (**Figure 1C** and **Supplementary Figure 1B**) and immunohistochemistry (**Figure 1D** and **Supplementary Figure 1C**).

### Heparanase Promotes Gastritis Inflammation and *H. pylori* Colonization

**Figure 2:**
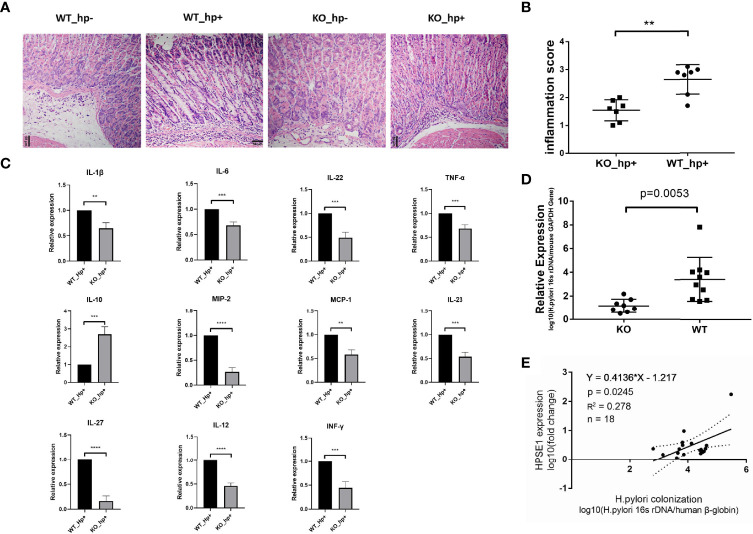
Heparanase facilitates the infiltration of macrophages in a model of chronic gastritis. WT and Hpa-KO mice were inoculated with *H. pylori* as described in **Figure 1**. Gastric tissues were harvested after 8 weeks and 5-micron sections were subjected to H&E staining **(A)**. The inflammatory score, evaluated by two independent expert pathologists, is shown in **(B)**. Total RNA was extracted from another portion of the tissues (WT mice n = 3, KO mice n = 3) and subjected to qPCR analyses applying primer sets specific for the indicated cytokine **(C)**. **(D)** Colonization of *H. pylori* in WT and Hpa-KO gastric tissue (WT mice n = 7, KO mice n = 7). The relative amounts of the 16S rRNA gene of *H. pylori* and the mouse beta-globin gene were quantified by qPCR. Note the low abundance of *H. pylori* in chronic gastritis of Hpa-KO mice (WT mice n = 10, KO mice n = 8). **(E)** Correlation between heparanase expression and the colonization of *H. pylori* in human samples. Human chronic gastritis and intestinal metaplasia tissues infected with *H. pylori* were collected during gastroscopy examination (chronic gastritis n = 11, intestinal metaplasia n = 7). One portion of the gastric mucosal tissue was used to extract the total DNA. The relative amounts of the 16S rRNA gene of *H. pylori* and the human beta-globin gene were quantified by qPCR. The other portion of the gastric mucosal tissue was used to extract the total RNA. The relative expression of human heparanase mRNA and human beta-globin mRNA was determined by qRT-PCR. **p <0.01; ***p <=0.001; ****p <=0.0001.

To investigate the role of heparanase in chronic gastritis, we infected Hpa-KO and Wild Type (WT) C57BL/6 mice with *H. pylori*, and the degree of inflammation in the gastric mucosa was examined after 8 weeks. As shown in **Figure 2A**, infiltration of immunocytes into the gastric mucosa of Hpa-KO mice and the degree of inflammation were significantly decreased as compared to control WT mice infected with *H. pylori*, consistent with the inflammatory score of the tissues (**Figure 2B**). Moreover, the expression of pro-inflammatory cytokines and chemokines including IL-1β, IL-6, IL-22, TNF-α, MIP-2, MCP-1, IL-23, IL-27, IL-12, and INF-γ was significantly decreased in Hpa-KO *vs* control WT mice, whereas the expression of IL-10, one of the main anti-inflammatory cytokines, was significantly increased in the Hpa-KO mice (**Figure 2C** and **Supplementary Figure 2**). To evaluate the impact of heparanase on the colonization of *H. pylori*, total DNA was extracted from the gastric mucosa, and the relative expression levels of *H. pylori* 16s rDNA were quantified. As shown in **Figure 2D**, *H. pylori* colonization was markedly decreased in gastric tissue of *H. pylori*-infected Hpa-KO *vs* WT mice, indicating that heparanase not only enhances gastric inflammation but also colonization of the bacteria. To study the effect of heparanase on the colonization of *H. pylori* in human chronic gastritis, patients with *H. pylori* infection were recruited and chronic gastritis tissues were collected during the gastroscopic examination. The collected gastric mucosa was divided into two parts and used to extract DNA and total RNA. The expression of human heparanase was quantified (RT-qPCR) relative to the 16S rRNA of *H. pylori* and the human beta-globin gene. Importantly, a positive correlation between expression of heparanase and colonization of *H. pylori* was found in human chronic gastritis (**Figure 2E**), further confirming the results of our mouse model (**Figure 2D**).

### Heparanase Facilitates Infiltration of Macrophages in *H. pylori*-Infected Chronic Gastritis

**Figure 3:**
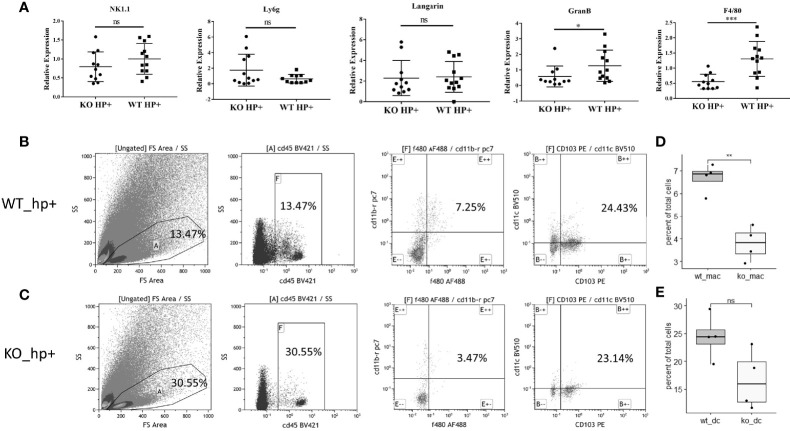
Reduced infiltration of macrophages into chronic gastritis tissue elicited by *H. pylori* in Hpa-KO mice. **(A)** qPCR. Chronic gastritis was elicited by *H. pylori* in WT and Hpa-KO mice (WT mice n = 12, KO mice n = 12), as described in **Figure 1**. Gastric tissues were collected after 8 weeks, total RNA was extracted and subjected to qPCR applying primer sets specific for NK1.1 and GranB (NK cells), Langarin (dendritic cells), F4/80 (macrophages), and Ly6g (neutrophils). Note, reduced infiltration of macrophages to the inflamed gastric tissue of Hpa-KO mice. **(B–E)** Flow cytometry. Fresh mucosa tissue (WT mice n = 4, KO mice n = 4), derived from *H. pylori*-infected chronic gastritis in WT and Hpa-KO mice, was dissociated into a single-cell suspension as described under *Materials and Methods*. Cells were then subjected to flow cytometry applying antibodies directed against CD45, F4/80, CD103, CD11b, and CD11c. The number of F4/80^+^CD11b^+^ macrophages and CD103^+^CD11c^+^ dendritic cells in WT *vs.* HPA-KO mice was compared. *p <0.05; **p <0.01; ***p <=0.001; ns, no significant difference.

To evaluate the impact of heparanase on the infiltration of immunocytes into the lamina propria of *H. pylori*-infected chronic gastritis mucosa, we evaluated (RT-qPCR) the expression of markers specific for the different immune cell populations, including NK1.1 and GranB (NK cells), Langarin (dendritic cells), F4/80 (macrophages), and Ly6g (neutrophils). We found that the recruitment of NK cells, dendritic cells, and neutrophils to the inflamed gastric tissue was comparable in WT and Hpa-KO mice (**Figure 3A**, left four panels). In striking contrast, recruitment of macrophages (evident by expression of F4/80) to the inflamed Hpa-KO gastric tissue was significantly and markedly decreased (**Figure 3A**, right panel). To further confirm the difference in macrophage infiltration, flow cytometry was applied by using fresh mucosa tissue of *H. pylori*-infected chronic gastritis derived from WT and Hpa-KO mice. Notably, the percentage of F4/80^+^CD11b^+^ macrophages in the gastritis mucosa of WT mice was significantly higher than that of Hpa-KO mice (**Figures 3B, D**, and **Supplementary Figure 4**). In contrast, the recruitment of CD103^+^CD11c^+^ dendritic cells appeared comparable in the gastritis mucosa of WT and Hpa-KO mice (**Figures 3C, E** and **Supplementary Figure 4**). Collectively, both the qPCR and flow cytometry analyses imply that heparanase facilitates the infiltration of macrophages in *H. pylori*-infected chronic gastritis.

### *H. pylori* Induces Expression of Heparanase in Gastric Epithelial Cells and Macrophages

**Figure 4:**
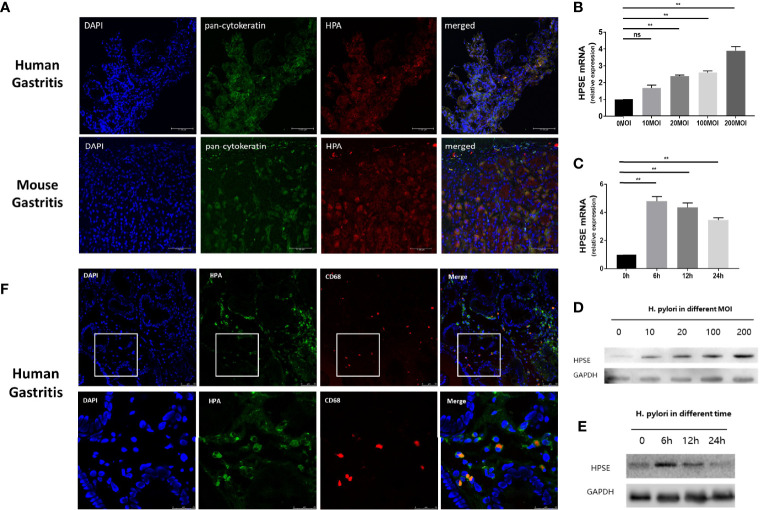
*H. pylori* induce the expression of heparanase in gastric epithelial cells and macrophages. **(A)** Immunofluorescent staining. *H. pylori*-positive human (upper panels) and mouse (lower panels) gastric tissue were collected and 5-micron sections were subjected to immunofluorescent staining applying anti-pan-cytokeratin (AE1/AE3, green) and anti-heparanase (red) antibodies. Images merged with nuclear staining (DAPI, blue) are shown in the right panels. Blue: cell nuclei; Green: anti-pan-cytokeratin monoclonal antibody, Alexa fluor 488; Red: primary, anti-heparanase antibody 733; secondary, cy3-labeled Goat Anti-Rabbit IgG antibody. **(B, C)** qPCR. *H. pylori* strain PMSS1 were co-cultured with GES-1 normal human gastric mucosa epithelial cells (n = 3 for each MOI) at the indicated multiplicity of infection **(B**, MOI**)** for 6 h. Total RNA was then extracted and subjected to qPCR applying a primer set specific for heparanase. Heparanase levels are shown graphically relative to its expression in control cells without the bacteria (0), set arbitrarily to a value of 1. Cells were similarly grown with *H. pylori* (200 MOI) for the indicated time (n = 3 for each time point) and heparanase expression was quantified by qPCR **(C)**. **(D, E)** Immunoblotting. GES-1 normal human gastric mucosa epithelial cells were similarly grown with *H. pylori* at the indicated MOI for 6 h. Cell extracts were then prepared and subjected to immunoblotting applying anti-heparanase (upper panel) and anti-GAPDH (lower panels) antibodies **(D)**. GES-1 cells were similarly grown with the bacteria (200 MOI) for the time indicated and cell lysates were subjected to immunoblotting as above **(E)**. **(F)** *H. pylori*-positive human gastric tissue samples were collected and 5-micron sections were subjected to immunofluorescent staining applying anti-heparanase (green) and anti-human CD68 (red, Alexa Fluor 647) antibodies. Merged images and nuclear staining (DAPI, blue) are shown in the right panels. **p <0.01; ns, no significant difference.

Heparanase is highly expressed in chronic gastritis with *H. pylori* infection, but the source of heparanase in gastritis tissue is still unclear. To investigate whether *H. pylori* induces the expression of heparanase in epithelial cells of the gastric mucosa, human and mouse gastritis tissues were double-stained with anti-pan-cytokeratin, a marker of epithelial cells, and anti-heparanase antibodies. As shown in **Figure 4A**, heparanase and cytokeratins are co-expressed in mouse and human gastritis tissues, consistent with the pattern of heparanase expression in other inflamed tissues. Moreover, the addition of *H. pylori* to normal human gastric epithelial cells (GES-1) resulted in increased heparanase expression and this increase appeared dose-dependent (**Figure 4B**). Notably, increased heparanase expression (over 5-folds) was evident already 6 h after the addition of the bacteria (**Figure 4C**) and was further confirmed by immunoblotting (**Figures 4D, E** **)**. Since heparanase facilitated the infiltration of macrophages in *H. pylori*-infected chronic gastritis (**Figure 3**), we stained *H. pylori*-infected human gastritis tissue for CD68 and heparanase. As shown in **Figure 4F**, heparanase staining co-localized with CD68^+^ macrophage staining, suggesting that heparanase in human gastritis originates also from macrophages.

### Heparanase Promotes Polarization of Macrophages to the M1 Phenotype

**Figure 5:**
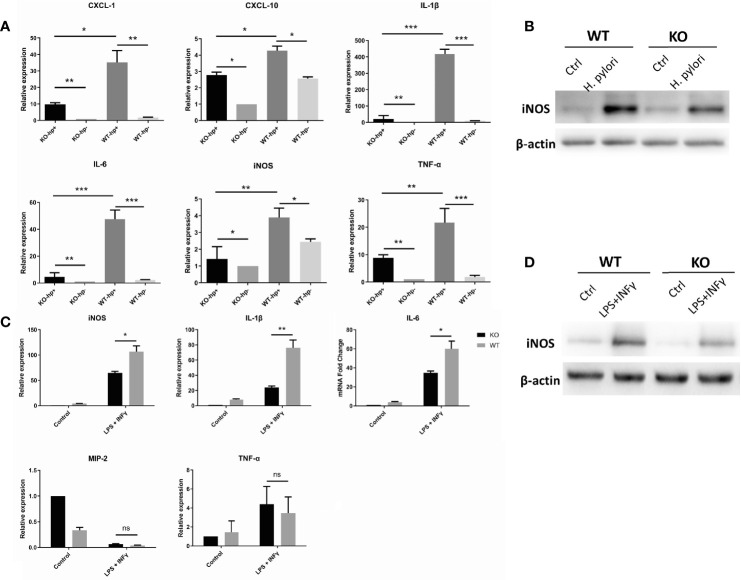
Heparanase promotes the polarization of macrophages to the M1 phenotype. **(A)** Peritoneal macrophages were isolated from WT and HPA-KO mice and were grown without or with *H. pylori* at 200 MOI for 6 h. Total RNA was then extracted, and the expression of IL-1β, IL-6, TNF-α, CXCL-1, CXCL-10 and iNOS was evaluated by qPCR. **(B, D)** Immunoblotting. Peritoneal macrophages were collected from WT and Hpa-KO mice and were left untreated (ctrl) or were treated with *H. pylori* **(**200 MOI; **B)**, or LPS (50 ng/ml) plus interferon γ **(**20 ng/ml**; D)** for 6 h. Cell extracts were then prepared and subjected to immunoblotting applying anti-iNOS (upper panels) and anti-actin (lower panels) antibodies. **(C)** qPCR. Macrophages were collected from the peritoneum of WT and Hpa-KO mice and were left untreated or treated with LPS plus interferon-gamma (IFN-γ; 20 ng/ml) for 6 h. Total RNA was then extracted and the relative expression of the iNOS, IL-1β, IL-6, TNF-α, MIP-2 and TNF-α was quantified by qPCR. *p <0.05; **p <0.01; ***p <=0.001; ns, no significant difference. KO-hp^+^: n = 3, KO-hp^-^: n = 3, WT-hp^+^: n = 3, WT-hp^-^: n = 3; KO ^control^: n = 3, KO^LPS + INF-γ^: n = 3, WT^control^: n = 3, WT-hp^LPS + INF-γ^: n = 3.

Macrophages, like other immune effector cells, have multiple subtypes and various phenotypes depending on the microenvironment. Specifically, macrophages can differentiate into distinct entities classically referred to as activated or inflammatory (M1) macrophages, and selectively activated or anti-inflammatory (M2) macrophages (25). The process of macrophage transformation from one phenotype to another is referred to as macrophage polarization (26). Importantly, M1 phenotype can be induced by IFN-γ, and bacterial lipopolysaccharide (LPS) (25). To evaluate the impact of heparanase on macrophage polarization, peritoneal macrophages were isolated from WT and Hpa-KO mice, and macrophages were maintained with 200 MOI *H. pylori* for 6 h. RNA was then extracted, and the expression of M1-related cytokines was determined by qPCR. Expression of IL-1β, IL-6, TNF-α, CXCL-1, CXCL-10 and iNOS in WT macrophages was higher in response to *H. pylori*, whereas a lower induction was quantified in Hpa-KO macrophages incubated with *H. pylori* (**Figure 5A** and **Supplementary Figure 5A**). This was also noted by immunoblotting for nitric oxide synthase (iNOS), a marker of M1 polarization (**Figure 5B**). We next performed a similar experiment except that macrophages were treated with LPS and IFN-γ instead of *H. pylori* (**Figure 5C**). The relative expression of IL-1β, IL-6, TNF-α, IL-10 and iNOS in macrophages from WT mice was significantly higher than in macrophages from Hpa-KO mice (**Figure 5C** and **Supplementary Figure 5B**), and this was further confirmed by immunoblotting for iNOS (**Figure 5D**). Moreover, whereas heparanase alone had no effect on iNOS expression, treatment of WT macrophages with heparanase and *H. pylori* resulted in a marked increase of iNOS expression, demonstrating a combined effect of *H. pylori* and heparanase on M1 polarization of macrophages.

### Heparanase-Stimulated M1 Polarization of Macrophages Involves p38 MAPK and NFκB

**Figure 6:**
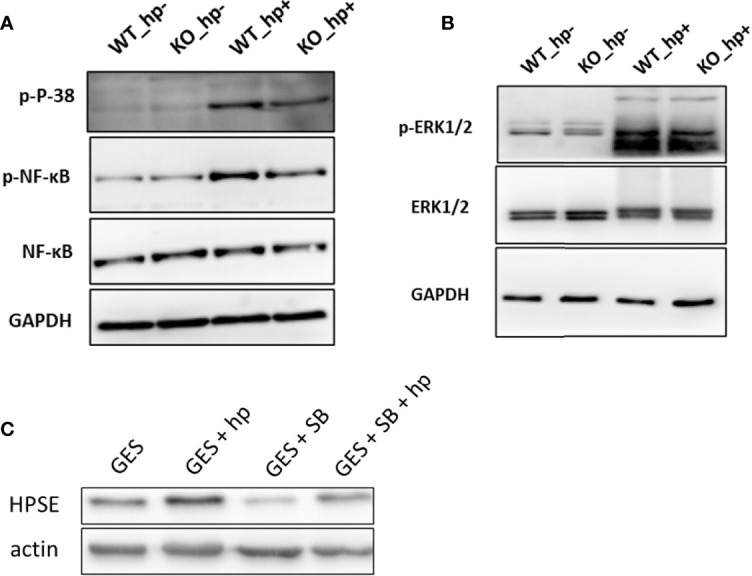
Stimulation of macrophage activation by *H. pylori* is associated with p38 MAPK and NF-κB phosphorylation. **(A)** Peritoneal macrophages were isolated from WT and Hpa-KO mice and inoculated without or with 200 MOI *H. pylori* for 6 h. Cell extracts were then prepared and subjected to immunoblotting applying anti-phospho-p38 (upper panel), anti-phospho-NFĸB (second panel), anti-total NF-ĸB (third panel) and anti-GAPDH (lower panel) antibodies. Cell extracts were similarly blotted with anti-phospho-Erk **(B**, upper panel**)**, anti-Erk **(B**, second panel**)** and anti-GAPDH **(B**, lower panel**)** antibodies **(C)** Heparanase expression. GES-1 cells were left untreated (GES) or were treated with *H. pylori* (200MOI; GES + *H. pylori*) without or with the p38 inhibitor SB203580 (20 μM; GES + SB + *H. pylori*) added 2 h before the bacteria. Cell extracts were prepared after 6 h and subjected to immunoblotting applying anti-heparanase (upper panel) and anti-actin (lower panel) antibodies.

Previous studies reported that heparanase is a key mediator of macrophage activation and function (23, 27–30). It was shown that heparanase activates Erk, p38, and JNK signaling in macrophages, leading to increased c-Fos levels and induction of cytokine expression (23). To explore whether the activation and M1 polarization of macrophages in *H. pylori*-infected gastritis utilize a similar mechanism, peritoneal macrophages were isolated from WT and Hpa-KO mice and inoculated with 200 MOI *H. pylori* for 6 h. Phosphorylation of Erk, MAPK, NF-κB and JNK was next examined by immunoblotting. As shown in **Figures 6A, B**, p38 MAPK and NFκB phosphorylation levels were increased by *H. pylori* in WT peritoneal macrophages whereas lower phosphorylation levels were detected in Hpa-KO macrophages. Interestingly, increased heparanase expression in GES-1 cells by *H. pylori* (**Figure 6C**) also involved p38 phosphorylation. This was concluded because heparanase induction by *H. pylori* was decreased markedly in GES-1 cells that were pre-treated with an inhibitor of p38 (SB203580; **Figure 6C**). This confirms previous reports that connect the p38 MAPK pathway with heparanase expression in gastric cancer cells (31).

## Discussion

In most tumor types, heparanase was reported to be overexpressed and correlated with increased tumor size, angiogenesis, metastasis and poor prognosis (8, 32–37). In line with the notion that heparanase is a drug target, three inhibitors of the heparanase enzyme (Roneparstat, Necuparanib, Pixatimod) have been evaluated in early-stage clinical trials, showing signs of efficacy (38–41). In addition, heparanase is capable of regulating multiple aspects of the inflammatory process (8, 11). Heparanase was reported to facilitate inflammation through the release of cytokines/chemokines anchored in the ECM, activation of innate immune cells and stimulation of cell motility and extravasation (8, 42–44). For example, heparanase was shown to promote ulcerative colitis (29), acute pancreatitis (14, 15), acute vasculitis (16), acute glomerulonephritis (17), sepsis (19), and hypersensitive pneumonia (18).

It has been previously reported that heparanase induced by *H. pylori* infection facilitates the proliferation, invasion and metastasis of gastric cancer cells (31, 45). However, the role of heparanase in gastritis and the early stages of gastric cancer initiation is still obscure. Here, we examined the involvement of heparanase in *H. pylori*-induced gastritis, which, test gastritis tissues and established a mouse model of *H. pylori*-infected chronic gastritis Expression and tissue localization of heparanase were illustrated by immunofluorescence and immunohistochemistry. Compared to normal gastric tissue, heparanase was overexpressed in *H. pylori*-infected chronic gastritis and intestinal metaplasia, indicating that high expression of heparanase is involved in bacteria-induced inflammation.

The regulation of heparanase in inflammation is complex and versatile (8). To investigate the specific function of heparanase in *H. pylori*-infected gastritis, we employed heparanase knockout (HPA-KO) C57BL/6 mice (20). Compared to wild-type (WT) C57BL/6 mice, deficiency of heparanase reduced the infiltration of immunocytes into the *lamina propria* of the gastric mucosa. The inflammation score of *H. pylori*-infected gastric mucosa derived from WT mice was significantly higher than that observed in heparanase knockout mice. Likewise, expression of pro-inflammatory cytokines (IL-1β, IL-6, TNF-α, IL-23, MIP-2, MCP-1, IL-27, IL-12, INF-γ) was significantly higher in WT *vs.* HPA-KO *H. pylori*-infected mice. These results indicate that heparanase plays an important role in *H. pylori*-induced chronic gastritis, corroborating previous studies on the involvement of MMP10 in gastritis and colonization of *H. pylori* (7). In contrast, MMP7 was reported to restrain *H. pylori*-induced gastric inflammation and premalignant lesions in the stomach by altering macrophage polarization (46). To explore the impact of heparanase on the colonization of *H. pylori*, Hpa-KO and WT mice were subjected to infection with *H. pylori*. Compared with WT mice, expression of the *H. pylori* 16s rRNA gene was significantly decreased in the gastric tissue of Hpa-KO mice. Likewise, a positive correlation was found between heparanase expression and *H. pylori* colonization in human gastric tissues. These results substantiate the notion that *H. pylori* promote high expression of heparanase which further facilitates the colonization of *H. pylori* in the gastric tissue. Notably, it was recently reported that *H. pylori* induces high expression of Rev-erbα that fosters the colonization of *H. pylori* by impairing host innate and adaptive defense (47), forming a positive feedback loop that aggravates *H. pylori* induced-gastritis. It appears that, among other effects, *H. pylori* promotes gastritis and gastric tumorigenesis *via* upregulated expression of heparanase (31), MMP10 (7) and Rev-erbα (47). Yet, the mechanism by which heparanase promotes colonization of *H. pylori* is still unknown and needs further investigation.

While the participation of heparanase in immunocyte chemotaxis, recruitment, extravasation, migration and accumulation in target inflammatory sites, is well documented (8, 10–13, 17, 19, 42, 44, 48, 49), its impact on immunocytes in *H. pylori*-induced chronic gastritis has not been analyzed. Our results indicate that heparanase regulates primarily the recruitment and accumulation of macrophages in *H. pylori*-induced chronic gastritis tissue, in agreement with previous studies on the impact of heparanase on macrophage recruitment and activation in cancer and inflammation (23, 27–30). Given that dendritic cells (DC) and IL-23 take part in the pathogenesis of *H. pylori*-induced gastritis (50), we examined the accumulation of DC in *H. pylori*-induced chronic gastritis. While decreased amount of DC was noted in *H. pylori*-infected Hpa-KO *vs* WT mice, this decrease was not statistically significant, further emphasizing the preferential involvement of macrophages in the observed heparanase-*H. Pylori* axis. Notably, Zhuang et al., proposed a multistep model of inflammation during H. pylori infection involving interactions between H. pylori, Th22 cells, DCs, gastric epithelial cells and myeloid-derived suppressor cells within the gastric mucosa (50). In other inflammatory settings, such as colitis (29), delayed-type hypersensitivity (51), inflammatory bowel disease (52) and lung injury caused by sepsis (53), overexpression of heparanase was primarily noted in epithelial and endothelial cells (54). Likewise, our results indicate that the main cellular source of heparanase in *H. pylori*-induced chronic gastritis are epithelial cells of the gastric mucosa, yet overexpression of heparanase in macrophages was also observed. Our results indicate that heparanase promotes M1 polarization of macrophages driven by *H. pylori* or LPS + INF-γ, resulting in increased expression of pro-inflammatory cytokines such as IL-1β, IL-6 and TNF-α, and further aggravating the severity of gastritis. While the underlying signaling mechanism needs to be elucidated, we have noted that heparanase facilitates M1 polarization in response to *H. pylori* mainly *via* activation of the p38 MAPK and NF-κB signaling pathway. Combining the current and previous results (29), it appears that the molecular mechanism underlying the activation and polarization of WT, but not Hpa-KO, macrophages involves a linear cascade by which heparanase activates Erk, p38, and JNK signaling in macrophages, leading to increased c-Fos and NFκB transcriptional activity and induction of cytokine expression (23). We also found that the COMPASS complex (54, 55) is impaired in the absence of heparanase, resulting in decreased levels of WDR5 and H3K4 methylation in Hpa-KO *vs.* WT macrophages (27). It remains to be elucidated whether the currently observed *H. pylori*-heparanase axis involves WDR5 induction and H3K4 methylation, given their important epigenetic roles in the progression of various cancers (56) including gastric cancer (57).

It is well documented that chronic inflammatory conditions contribute to cancer progression (46, 47, 58–62) through, among other mechanisms, mobilization of tumor-supporting immunocytes (e.g., tumor-associated macrophages, neutrophils) which supply bioactive molecules that foster cell survival, angiogenesis, invasion and metastasis (42, 47, 48). Moreover, in several anatomic sites chronic inflammation is crucially implicated in tumor initiation, producing a mutagenic environment through the release of reactive oxygen/nitrogen species from infiltrating immune cells, generating cytokines, chemokines, growth factors, and anti-apoptotic proteins, and activating tumor-stimulating signaling pathways (e.g., NF-kB, p38MAPK, STAT3) (42, 47–49). Progression of Barrett’s esophagus to adenocarcinoma (63); chronic gastritis to intestinal-type gastric carcinoma (64, 65), chronic hepatitis C to hepatocellular carcinoma (64); pancreatitis to pancreatic adenocarcinoma (66) and colitis to colorectal cancer (67) are well-known examples of inflammation-driven tumorigenesis. Remarkably, induction of heparanase before the appearance of malignancy was reported in essentially all of the above-mentioned inflammatory conditions, i.e., Barrett’s esophagus (68), hepatitis C infection (69), chronic pancreatitis (70), Crohn disease and ulcerative colitis (29, 50). Similarly, *H. pylori*-induced gastric inflammation and the associated up-regulation of heparanase, observed in the current study, likely promote the development of gastric cancer.

Given the causal role of heparanase in tumor progression in tissues in which cancer-related inflammation typically occurs, (i.e., gastrointestinal tract, pancreas, liver), it is conceivable that inflammation-induced heparanase is involved in coupling inflammation and cancer. This notion is supported by a study utilizing a mouse model of colitis-associated colon carcinoma (29), showing that heparanase promotes polarization of innate immunocytes toward pro-inflammatory and/or pro-tumorigenic phenotype. The same self-sustaining crosstalk between the gastric epithelium and immunocytes appears to be driven by *H. pylori* infection. By sustaining continuous activation and polarization of macrophages that supply cancer-promoting cytokines (i.e., IL-1, IL-6, IL-1β, TNF-α, MIP-2, IL-10, iNOS), heparanase (produced primarily by inflamed epithelium) may participate in creating a pro-tumorigenic microenvironment, characterized by enhanced NFκB and p38-MAPK signaling (71), that supports the development and progression of gastric cancer.

In conclusion, our results indicate that *H. pylori* infection promotes overexpression of heparanase in gastritis which in turn facilitates the colonization of *H. pylori* and hence worsens gastritis. Inhibition of heparanase by heparin/HS mimicking compounds, neutralizing antibodies and/or small molecules may block this self-sustaining pro-inflammatory cycle and thereby reduce the risk of gastritis and the associated gastric cancer.

## Data Availability Statement

The original contributions presented in the study are included in the article/**Supplementary Material**. Further inquiries can be directed to the corresponding authors.

## Ethics Statement

The study was approved by the Ethics Committee of Xinqiao Hospital, The Army Medical University. Written informed consent was obtained from each individual. All the breeding processes and animal experiments were reviewed and approved by the Ethics Committee of Xinqiao Hospital, The Army Medical University.

## Author Contributions

Conceptualization and design: SY and IV. Experimental work: LT, BT, YYL, MY, SW, SH, ZX and YJL. Writing: Original Draft, LT and BT. Writing, Review and Editing: SY and IV. Supervision, SY and IV. All authors contributed to the article and approved the submitted version.

## Funding

This study was generously supported by a research grant awarded to SY and IV and by the ISF-NSFC joint research program [grant No. 2572/16 (Israel side) and No. 81661148050 (China side)]. It was also supported by grants from the Israel Science Foundation (1021/19) and the Israel Cancer Research Fund (ICRF), IV is a Research Professor of the Israel Cancer Research Fund (ICRF).

## Conflict of Interest

The authors declare that the research was conducted in the absence of any commercial or financial relationships that could be construed as a potential conflict of interest.

## Supplementary Material

Supplementary Figure 1 Semi-quantitative scoring of immunohistochemical and immunofluorescence results. **(A)** Semi-quantitative scoring of the immunofluorescence presented in **Figure 1A** (n=4 for each group). **(B)** Semi-quantitative scoring of immunofluorescence presented in Figure. 1C (n=4 for each group). **(C)** Semi-quantitative scoring of the immunohistochemical study presented in Figure 1D (n=4 for each group).

Supplementary Figure 2 ELISA quantification of all the pro‐inflammatory and pro‐tumorigenic cytokines presented in **Figure 2C**. WT Hp+: n=6, KO Hp+: n=5. *p<0.05. * p<0.05; **p<0.01; ***p<=0.001; ns: no significant difference.

Supplementary Figure 3 Culture and Gram staining of two *H. pylori*-positive samples. Gastric mucosa tissues were grinned into tissue homogenates and resuspended with sterile PBS. After centrifugation, the supernatant was added onto blood culture plate containing antibiotics and cultured in 5% O2,10% CO2 and 85% N2 incubator at 37°C for 48 hours. Left: Gray dots on the blood culture plate are colonies of *H. pylori.* Right: gram staining of *H. pylori*.

Supplementary Figure 4 FACS blanks for the detection of dendritic cells and macrophages using anti F4/80+, CD11b+, CD103+ and CD11c+ antibodies.

Supplementary Figure 5 ELISA quantification of cytokines. **(A)** ELISA quantification of the cytokines presented in **Figure 5A**. **(B)** ELISA quantification of the cytokines presented in **Figure 5C**. KO-hp+: n =6; KO-hp-: n =6; WT-hp+: n = 6; WT-hp-: n =3; KO control: n =6; KO ^LPS+INF-γ:^ n =6; WT ^control:^ n = 6; WT-hp ^LPS+INF-γ:^ n =6. *p<0.05; **p<0.01; *** p<=0.001; ns: no significant difference.
