# Supplementary figures and images for: Helicobacter pylori-Induced Heparanase Promotes H. pylori Colonization and Gastritis
Source: Front Immunol. 2021 Jun 17;12:675747. doi: 10.3389/fimmu.2021.675747 (PMC8248549; doi:10.3389/fimmu.2021.675747)

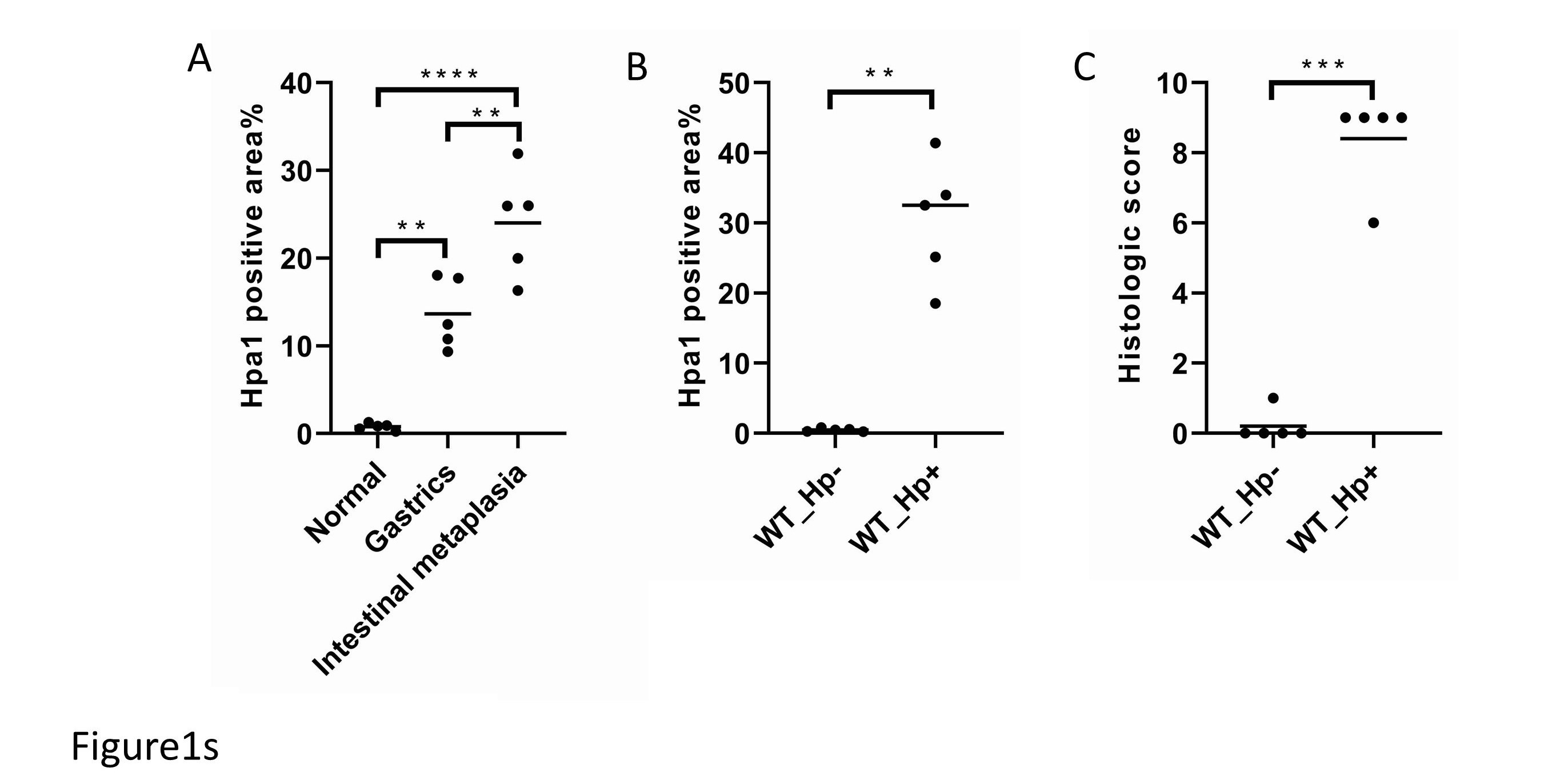

Supplement: Supplementary Figure 1 — Semi-quantitative scoring of immunohistochemical and immunofluorescence results. (A) Semi-quantitative scoring of the immunofluorescence presented in Figure 1A (n=4 for each group). (B) Semi-quantitative scoring of immunofluorescence presented in Figure. 1C (n=4 for each group). (C) Semi-quantitative scoring of the immunohistochemical study presented in Figure 1D (n=4 for each group). [file Image_1.jpeg]

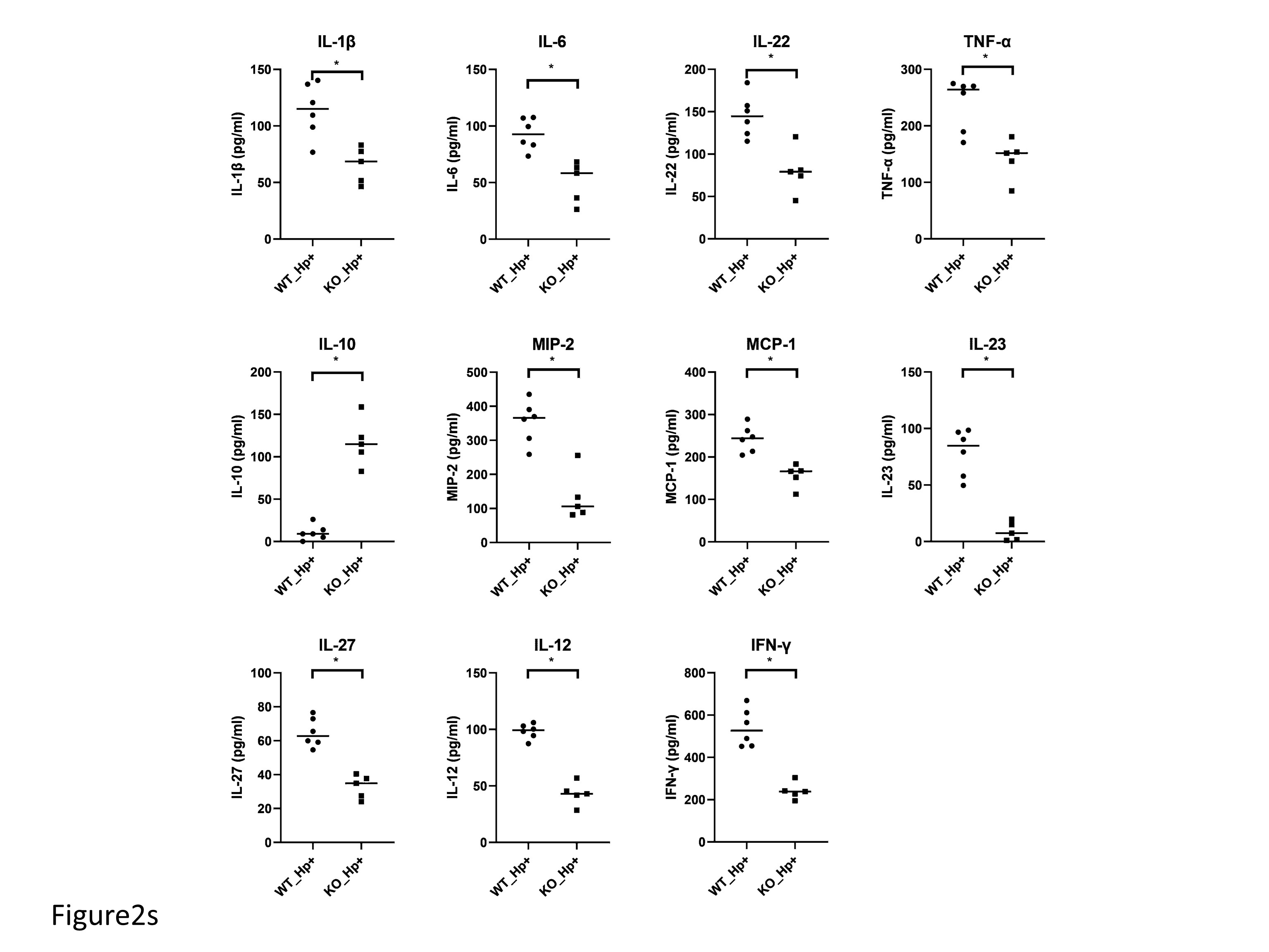

Supplement: Supplementary Figure 2 — ELISA quantification of all the pro‐inflammatory and pro‐tumorigenic cytokines presented in Figure 2C . WT Hp+: n=6, KO Hp+: n=5. *p<0.05. * p<0.05; **p<0.01; ***p<=0.001; ns: no significant difference. [file Image_2.jpeg]

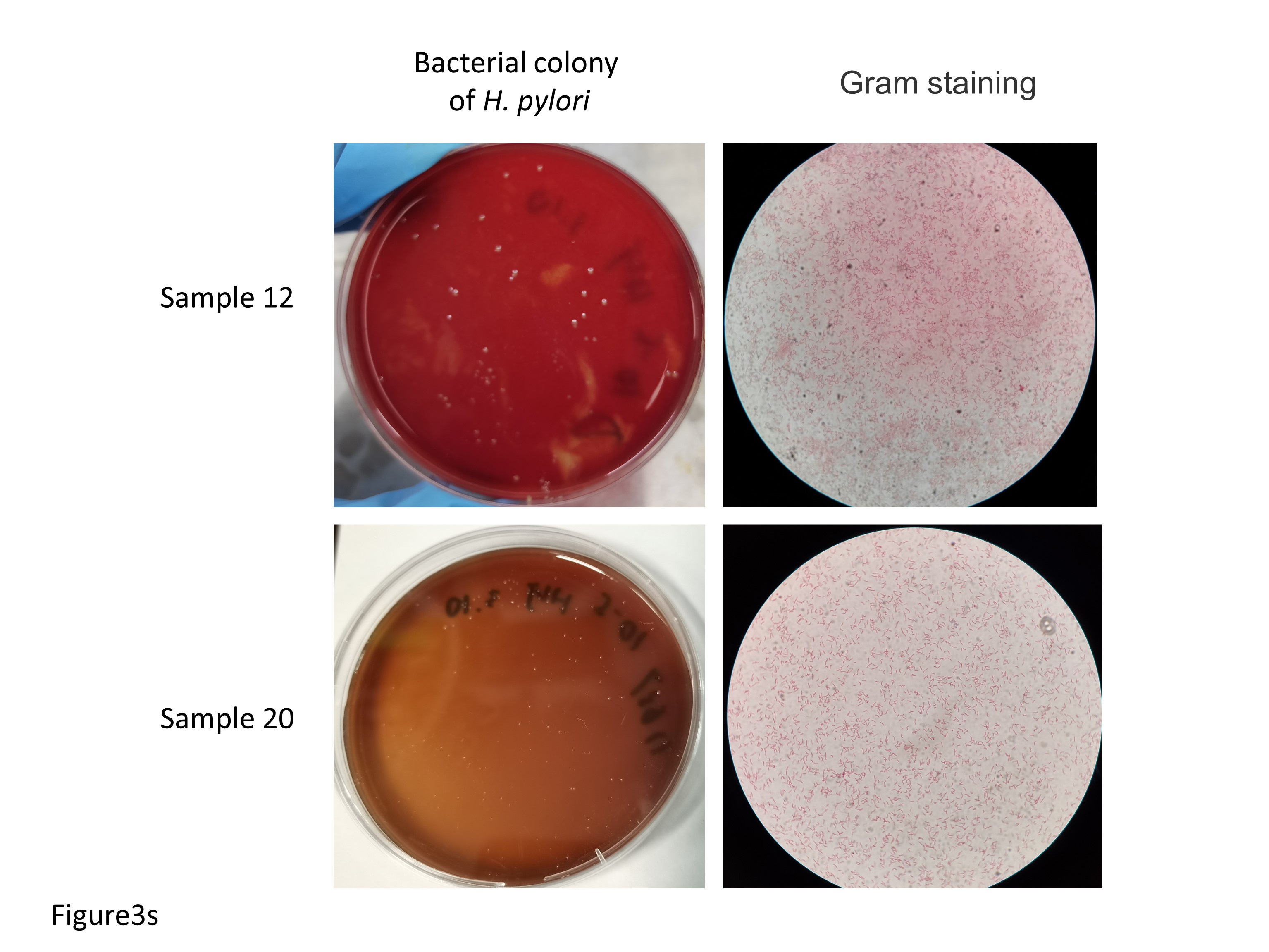

Supplement: Supplementary Figure 3 — Culture and Gram staining of two H. pylori-positive samples. Gastric mucosa tissues were grinned into tissue homogenates and resuspended with sterile PBS. After centrifugation, the supernatant was added onto blood culture plate containing antibiotics and cultured in 5% O2,10% CO2 and 85% N2 incubator at 37°C for 48 hours. Left: Gray dots on the blood culture plate are colonies of H. pylori. Right: gram staining of H. pylori. [file Image_3.jpeg]

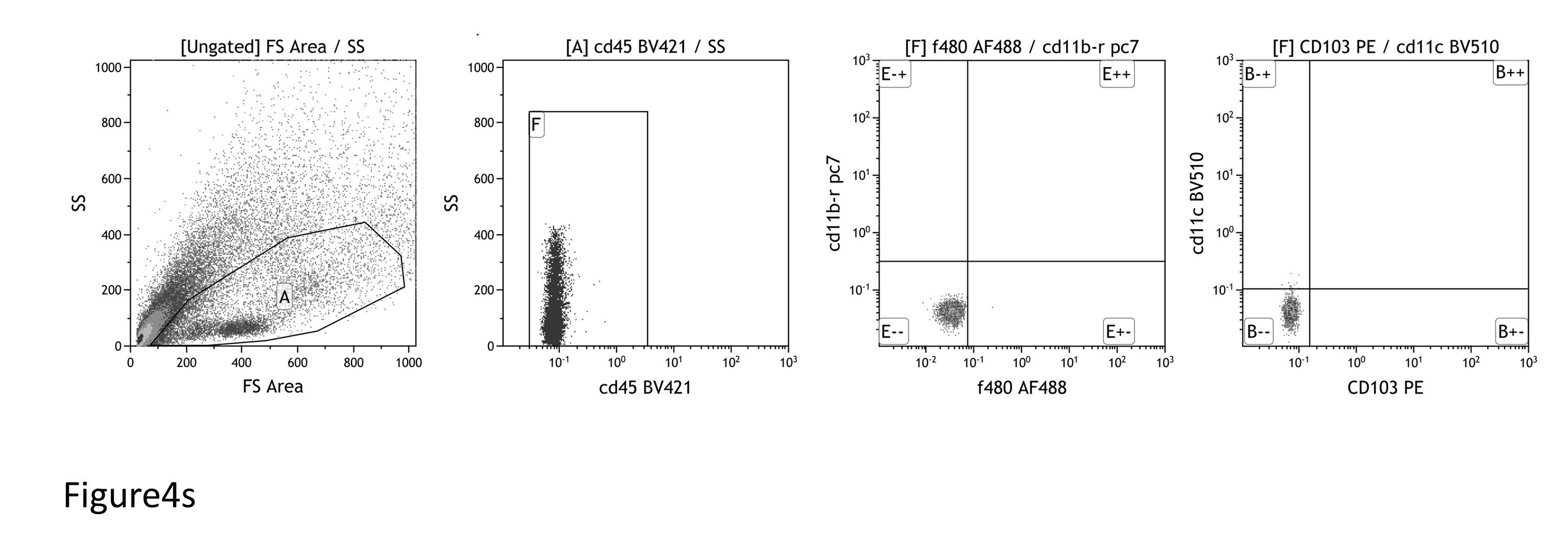

Supplement: Supplementary Figure 4 — FACS blanks for the detection of dendritic cells and macrophages using anti F4/80+, CD11b+, CD103+ and CD11c+ antibodies. [file Image_4.jpeg]

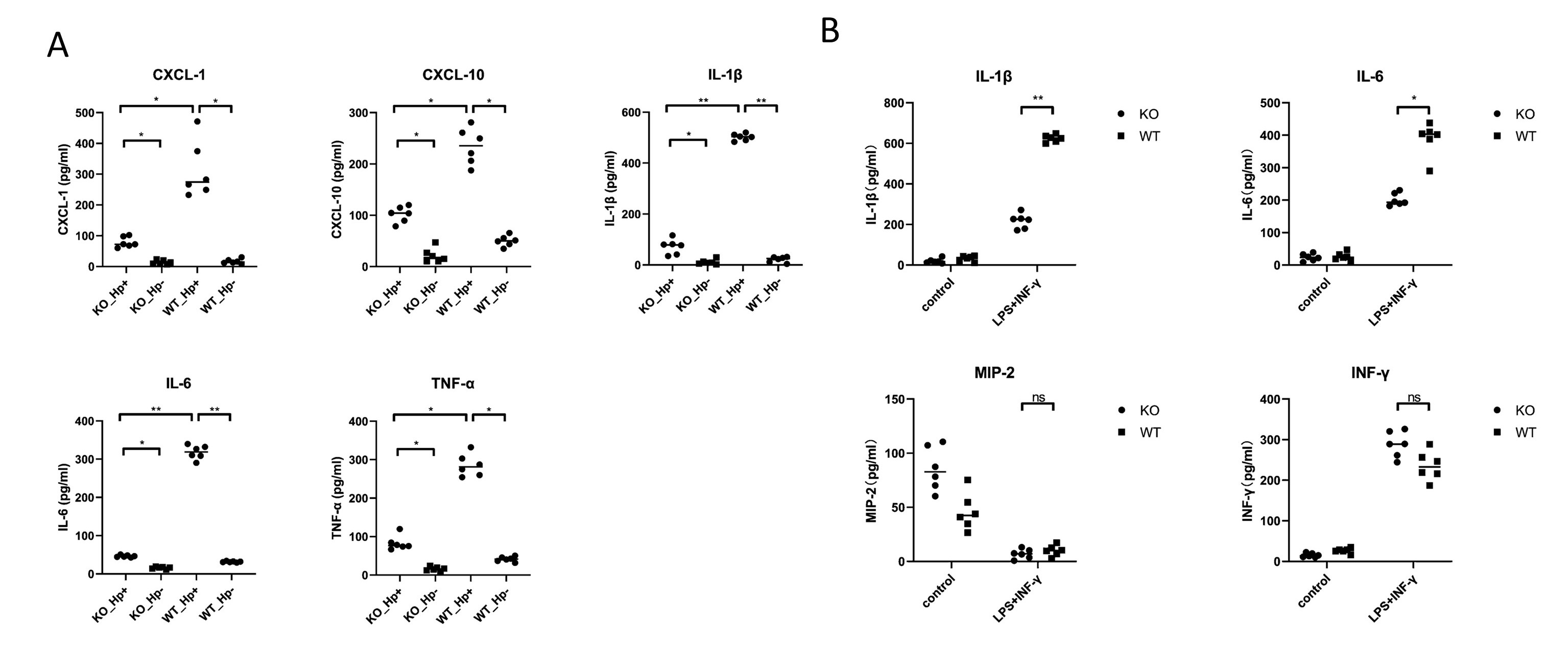

Supplement: Supplementary Figure 5 — ELISA quantification of cytokines. (A) ELISA quantification of the cytokines presented in Figure 5A . (B) ELISA quantification of the cytokines presented in Figure 5C . KO-hp+: n =6; KO-hp-: n =6; WT-hp+: n = 6; WT-hp-: n =3; KO control: n =6; KO LPS+INF-γ: n =6; WT control: n = 6; WT-hp LPS+INF-γ: n =6. *p<0.05; **p<0.01; *** p<=0.001; ns: no significant difference. [file Image_5.jpg]
