# Supplementary material for: Helicobacter pylori-Induced Heparanase Promotes H. pylori Colonization and Gastritis
Source: Front Immunol. 2021 Jun 17;12:675747. doi: 10.3389/fimmu.2021.675747 (PMC8248549; doi:10.3389/fimmu.2021.675747)
Supplement: Supplementary file 6 [file DataSheet_1.docx]

**Supplementary tables**

| **Supplementary Table 1. Primers for detecting H. pylori colonization by using cDNA templates** | |
| --- | --- |
| **Primer for DNA** | **Sequences** |
| H. pylori 16s rDNA F | 5`-TTTGTTAGAGAAGATAATGACGGTATCTAAC-3` |
| H. pylori 16s rDNA R | 5`-CATAGGATTTCACACCTGACTGACTATC-3` |
| H. pylori 16s rDNA Probe: | 5`-6-FAM-CGTGCCAGCAGCCGCGGT-TAMRA-N-3` |
| human-β-globin F | 5`-TGCCTATCAGAAAGTGGTGGCT-3` |
| human-β-globin R | 5`-GCTCAAGGCCCTTCATAATATCC-3` |
| human-β-globin Probe | 5`-6-FAM-TGGCTAATGCCCTGGCCCACAA-TAMRA-N-3` |
| mouse GAPDH-DNA F | 5`-TGCACCACCAACTGCTTAG-3` |
| mouse GAPDH-DNA R | 5`-GGATGCAGGGATGATGTTC-3` |
| mouse GAPDH-DNA probe | 5`-6-FAM-CAGAAGACTGTGGATGGCCCT-TAMRA-N-3` |

| **Supplementary Table 2. Primers for detecting mRNA by using cDNA templates** | | |
| --- | --- | --- |
| **Primer for mRNA** | **Sense 5’-3’** | **Anti-sense 5’-3’** |
| human HPSE | CGGCTAAGATGCTGAAGAGC | TGATGCCATGTAACTGAATCAA |
| human beta-actin | GCACTCTTCCAGCCTTCCTT | CGTACAGGTCTTTGCGGATG |
| mouse HPSE | GAGCGGAGCAAACTCCGAGTGTATC | GATCCAGAATTTGACCGTTCAGTT |
| mouse beta-actin | ATGCTCCCCGGGCTGTAT | CATAGGAGTCCTTCTGACCCATTC |
| mouse IL-1β | GAAGAAGAGCCCATCCTCTG | TCATCTCGGAGCCTGTAGTG |
| mouse iNOS | CAGCTGGGCTGTACAAACCTT | CATTGGAAGTGAAGCGTTTCG |
| mouse CXCL-10 | GCTGCAACTGCATCCATATC | TTTCATCGTGGCAATGATCT |
| mouse CXCL-1 | ACCCAAACCGAAGTCATAGC | GTGCCATCAGAGCAGTCTGT |
| mouse IL-6 | CTGCAAGAGACTTCCATCCAGTT | GAAGTAGGGAAGGCCGTGG |
| mouse TNF-α | CATCTTCTCAAAATTCGAGTGACAA | TGGGAGTAGACAAGGTACAACCC |
| mouse MIP-2 | CCACTCTCAAGGGCGGTCAAA | TACGATCCAGGCTTCCCGGGT |
| mouse MCP-1 | AGCAGGTGTCCCAAAGAAGC | ACAGAAGTGCTTGAGGTGGT |
| mouse IL-10 | CCAAGCCTTATCGGAAATGA | TCACTCTTCACCTGCTCCAC |
| mouse IL-22 | TCGCCTTGATCTCTCCACTC | GCTCAGCTCCTGTCACATCA |
| mouse IL-23 | TGCTGGATTGCAGAGCAGTAA | ATGCAGAGATTCCGAGAGA |
| mouse IL-27 | CTCTGCTTCCTCGCTACCAC | GGGGCAGCTTCTTTTCTTCT |
| mouse IL-12 | GAGGACTTGAAGATGTACAG | TTCTATCTGTGTGAGGAGGGC |
| mouse INF-γ | AGCTCTTCCTCATGGCTGTT | TTTGCCAGTTCCTCCAGATA |
| mouse NK1.1 | GCTGTGCTGGGCTCATCCT | TTGATGGTTTTTGTACTAAGACTCGCA |
| mouse Ly6g | TGCCCCTTCTCTGATGGATT | TGCTCTTGACTTTGCTTCTGTGA |
| mouse Langarin | GGACTACAGAACAGCTTGGAGAATG | TACTTCCAGCCTCGAGCCAC |
| mouse Granzyme B | TGTCTCTGGCCTCCAGGACAA | CTCAGGCTGCTGATCCTTGATCGA |
| mouse F4/80 | GATACAGCAATGCCAAGCAGT | TTGTGAAGGTAGCATTCACAAGTGTA |
